# Interorgan communication through peripherally derived peptide hormones in *Drosophila*

**DOI:** 10.1080/19336934.2022.2061834

**Published:** 2022-05-01

**Authors:** Naoki Okamoto, Akira Watanabe

**Affiliations:** a Life Science Center for Survival Dynamics, Tsukuba Advanced Research Alliance (TARA), University of Tsukuba, Tsukuba, Ibaraki, Japan; b Degree Programs in Life and Earth Sciences, Graduate School of Science and Technology, University of Tsukuba, Tsukuba, Ibaraki, Japan

**Keywords:** Interorgan communication, peripheral tissues, peptide hormones, ecdysone, juvenile hormone, innate behaviour, growth, metabolism, post-mating responses, reproduction

## Abstract

In multicellular organisms, endocrine factors such as hormones and cytokines regulate development and homoeostasis through communication between different organs. For understanding such interorgan communications through endocrine factors, the fruit fly *Drosophila melanogaster* serves as an excellent model system due to conservation of essential endocrine systems between flies and mammals and availability of powerful genetic tools. In *Drosophila* and other insects, functions of neuropeptides or peptide hormones from the central nervous system have been extensively studied. However, a series of recent studies conducted in *Drosophila* revealed that peptide hormones derived from peripheral tissues also play critical roles in regulating multiple biological processes, including growth, metabolism, reproduction, and behaviour. Here, we summarise recent advances in understanding target organs/tissues and functions of peripherally derived peptide hormones in *Drosophila* and describe how these hormones contribute to various biological events through interorgan communications.

## Introduction

More than a century has passed since the discovery of insulin [1], and it is now evident that organs coordinate their physiological states through various endocrine factors, including hormones and cytokines. Among them, peptide hormones play central roles in regulating multiple developmental and physiological processes, such as growth, metabolism, reproduction, and behaviour in animals, including mammals and insects [2,3]. Although neuropeptides act as modulators of neural activity in the central nervous system (CNS), they also function as peptide hormones secreted into circulation and act on various organs and tissues. Similarly, peptide hormones produced in peripheral tissues function as endocrine factors that mediate communications between multiple organs to coordinate their physiological states through the circulatory system.

Although insects have open vascular systems, peptide hormone-mediated endocrine pathways have multiple levels of similarities between insects and mammals [4], as exemplified by conservation of the insulin/insulin-like growth factor (IGF) system [5–7]. Moreover, applications of the tissue- or cell-specific molecular genetic tools in the fruit fly *Drosophila melanogaster* have primarily contributed to accumulating knowledge on endocrine factor-mediated interorgan communications [4]. In insects including *Drosophila*, as most peptide hormones, such as insulin-like peptides (ILPs), are mainly produced in the CNS, functions of neuropeptides and peptide hormones produced in the CNS have been extensively studied for many years [8–10]. Recently, however, it has become evident that different types of peptide hormones produced in peripheral tissues also play fundamental roles in critical biological events [4,11]. Here, we review a variety of peptide hormones produced in the peripheral tissues in *Drosophila* to understand their functions in regulating multiple developmental and physiological processes, such as growth, metabolism, reproduction, and behaviour.

In order to sort out issues to be addressed in this review, we first need to define the difference between neuropeptides and peptide hormones. Neuropeptides are peptides produced and released by neurons and act on neural substrates. In other words, the only difference between neuropeptides and peptide hormones produced in the CNS is whether they act within the CNS or act on peripheral tissues. Therefore, most neuropeptides also function as peptide hormones and *vice versa*. In this review, the term ‘neuropeptides’ refers to peptides produced in the CNS that act within the CNS, whereas the term ‘peptide hormones’ refer to peptides produced in the CNS and acting on peripheral tissues, or those produced in peripheral tissues that act on other organs or tissues. This review also defines peptide hormones as secreted proteins of approximately 100 amino acid residues or less. Please refer to other recent reviews for larger secreted proteins, cytokines, or molecules not encoded by genes that act as hormones in *Drosophila* [4,12,13]. This review also excludes topics related to antimicrobial peptides [14]. Finally, sex peptide (SP) cannot be strictly defined as a peptide hormone, but since it acts as a peptide hormone after being transferred from males to females, we cover SP in this review.

## 1. Peripherally derived peptide hormones: ecdysone biosynthesis

Moulting and metamorphosis in insects are primarily regulated by the steroid hormone called ecdysone (more specifically, its biologically active form 20-hydroxyecdysone or 20E) [15–17]. Ecdysone is synthesised in the specialised steroidogenic organ called the prothoracic gland(s) (PG) [18–21], and it regulates expression of various genes involved in moulting and metamorphosis [22–24]. Previous insect physiology studies have characterised many peptide hormones and other factors that regulate ecdysone production in the PG. As insect developmental events such as moulting, pupation, and eclosion are highly dependent on environmental signals processed in the brain, most peptide hormones and other factors regulating ecdysone biosynthesis are produced in the CNS [13,21,25–27].

The prothoracicotropic hormone (PTTH), originally called the ‘brain hormone’ [28–30], is a primary trophic factor responsible for ecdysone production in the PG [31,32]. PTTH is secreted from two pairs of neurosecretory cells in the CNS and acts on PG either through circulation in lepidopteran insects [33–36] or through direct neuronal innervation in *Drosophila* [32]. PTTH binds to a receptor tyrosine kinase (RTK) called Torso in the PG and activates ERK signalling pathway to stimulate ecdysone production [37]. In recent years, more evidence has accumulated that multiple peptide hormones or other factors act on the PG or PTTH-producing neurons to control ecdysone production [13,21,26,27]. In addition to PTTH, ILPs are essential factors from the CNS that promote ecdysone production in the PG [38–43]. The *Drosophila* genome contains eight ILP (Dilp) genes called *dilp1-dilp8* encoding peptide hormones of the insulin/IGF/relaxin family [7,44,45]. The major Dilps (Dilp2, −3, and −5) are produced mainly in a cluster of neurosecretory cells called insulin-producing cells (IPCs) in the *pars intercerebralis* (PI) region of the brain [44–47]. Secretion of Dilps from IPCs is tightly regulated by nutritional status, and Dilps bind to an RTK named insulin-like receptor (InR) to activate insulin/IGF signalling in many target tissues including the PG (please refer to the nutritional regulation of Dilp release from the brain IPCs in Section 3). Developmental and nutritional inputs from the CNS to the PG are thus mainly mediated by PTTH and Dilps, respectively, which regulate ecdysone production in *Drosophila*.

**Figure 1.**
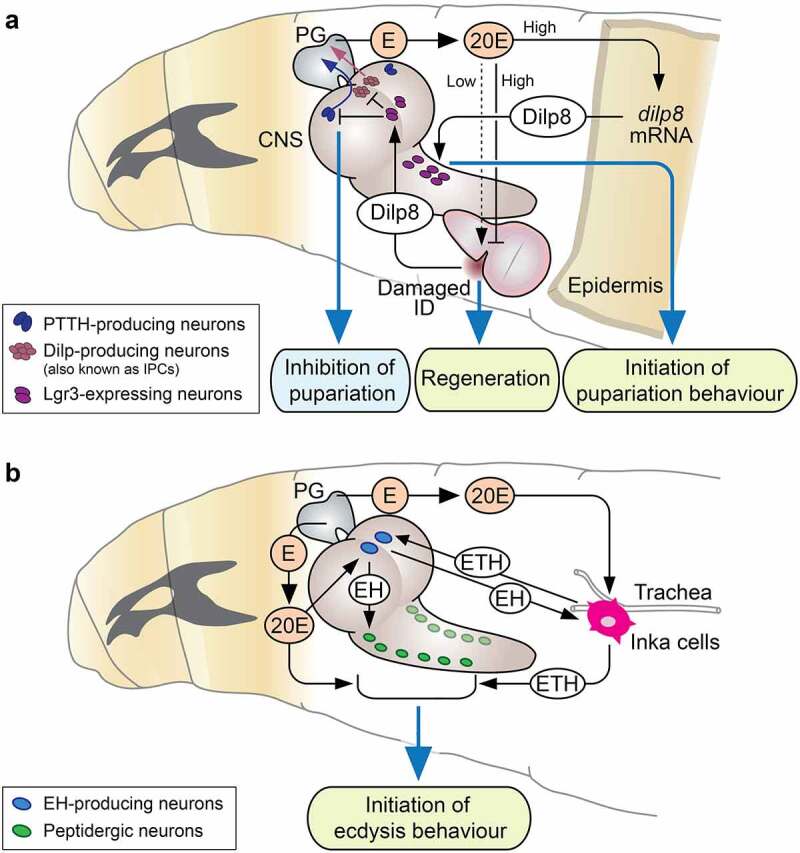
Interorgan communication by peripherally derived peptide hormones regulating ecdysone-related developmental processes. (a) Systemic functions of *Drosophila* insulin-like peptide 8 (Dilp8) from the damaged imaginal disks (ID) or epidermis during development. Dilp8 from the damaged ID delays the onset of pupariation by antagonising ecdysone production. The lower concentrations of 20-hydroxyecdysone (20E, a biologically active ecdysone), suppressed by Dilp8, effectively promote regenerative activity in damaged ID, whereas higher concentrations suppress regeneration. At the onset of pupariation, the higher concentrations of 20E triggers transient expression and secretion of Dilp8 from epidermis, which in turn promotes pupariation behaviour. (b) Systemic function of ecdysis-triggering hormone (ETH) from Inka cells that triggers ecdysis behaviour in response to 20E. ETH remotely acts on ETH receptor (ETHR) in multiple peptidergic neurons in the CNS, including eclosion hormone (EH)-producing neurons, which sequentially induces ecdysis behaviour. Abbreviations; E, ecdysone (an immediate precursor of a biologically active ecdysone); PG, prothoracic gland; CNS, central nervous system; PTTH, prothoracicotropic hormone; Lgr3, leucine-rich repeat-containing G protein-coupled receptor 3.

Interestingly, a series of recent studies have shown that a peripherally derived peptide hormone of the insulin/IGF/relaxin family called Dilp8 acts upstream of ecdysone production to control developmental timing. Although the major Dilps are produced mainly in the brain IPCs, IGF-like Dilp6 and relaxin-like Dilp8 are produced mainly in peripheral tissues [48–52] (function of Dilp6 is described in Section 3). Dilp8 is released from damaged or wounded imaginal disks to delay the timing of puparium formation during larval development [50,51]. Secreted Dilp8 acts on leucine-rich repeat-containing G protein-coupled receptor (GPCR) 3 (Lgr3), which is a member of the highly conserved family of relaxin family peptide receptors, expressed in the CNS [53–55] (Figure 1a). Two pairs of Lgr3-expressing neurons in the PI region of the brain make direct contact with PTTH-producing neurons and IPCs to inhibit PTTH and Dilp production, respectively [53–55], thereby preventing the ecdysone surge that initiates pupariation (Figure 1a). Lgr3 is also required in the PG itself for regulating ecdysone production during regeneration [56]. Notably, the lower concentrations of ecdysone, suppressed by Dilp8, effectively promote regenerative activity in damaged imaginal disks, whereas higher concentrations suppress regeneration [57] (Figure 1a). This peripheral tissue-CNS interaction is a typical mechanism to ensure developmental robustness in multicellular organisms, as it secures enough time for regenerating damaged imaginal disks before pupariation.

In addition to Dilp8, factors, such as the TGF-β decapentaplegic (Dpp), Hedgehog (Hh), and cytokine Unpaired-3 (Upd3) from peripheral tissues remotely regulate ecdysone production and regulate initiation of metamorphosis [58–61]. Dpp and HH are recognised as morphogens that play a central role in tissue morphogenesis in *Drosophila*, but these morphogens also act as endocrine hormones, playing integral physiological roles in interorgan communication.

Several factors that suppress the steroidogenic function of the PG have been reported in many insects [62–75]. Among them, the prothoracicostatic peptide (PTSP) discovered in the silkworm *Bombyx mori* is a peripherally derived peptide involved in suppressing ecdysone production in the PG [68,72,76,77]. PTSP, which belongs to the W(X)_6_Wamide peptide family, is also known as the myoinhibiting peptide (MIP)/allatostatin-B (AstB) in other insects, including *Drosophila* [68]. In *Bombyx*, PTSP is produced in the CNS and epiproctodeal gland (EPG), the latter of which is a group of peripheral endocrine cells located in the anterior part of the rectum [72,78]. Notably, the PTSP receptor (PTSPR), an orthologue of the *Drosophila* sex peptide receptor (SPR), is highly expressed in the PG just before each larval or pupal ecdysis in *Bombyx* [72]. Furthermore, temporal expression levels and immunoreactivity of PTSP in the EPG strongly correlate with the rapid drop in ecdysone titre just before ecdysis [72,78], suggesting that PTSP secreted from the EPG acts directly on PG to inhibit ecdysone production [72]. In *Drosophila*, MIP/AstB is produced by neurosecretory cells in the CNS and peptidergic enteroendocrine cells (EECs) in the gut [79–81], but to date, no studies have evaluated its role in ecdysone production.

## 2. Peripherally derived peptide hormones: innate behaviours during development

In addition to upstream factors of ecdysone production, several peripherally derived peptide hormones act downstream of ecdysone to coordinate ecdysone-dependent innate behaviours, such as ecdysis and pupariation.

Insect moulting is a series of highly complex physiological and behavioural processes regulated by ecdysone [82–84]. In the early phase of moulting, an increase in the level of ecdysone leads to apolysis (detachment of the cuticle from the epidermis), with the formation of a new cuticle layer deposited by the epidermis [85,86]. After a subsequent decrease in ecdysone levels, a peripherally derived peptide called ecdysis-triggering hormone (ETH) plays a central role in regulating ecdysis, a behavioural sequence that leads to the shedding of the old cuticle [83]. ETH is an amidated peptide produced by large peripheral endocrine cells called Inka cells, which form part of the epitracheal gland (EG) [83]. The EG is a group of endocrine glands attached to the main tracheal tube in each body segment in insects [87,88], and its physiological function has become evident with the identification and characterization of ETH in the tobacco hornworm *Manduca sexta* [89,90]. In *Drosophila, ETH* gene encodes two mature peptides, ETH-1 and ETH-2 [91]. *ETH* expression in Inka cells is induced by an increase in ecdysone levels during the early phase of moulting [90,92] (Figure 1b). Ecdysone simultaneously upregulates expression of the GPCR for ETH called the ETH receptor (ETHR) in various peptidergic neurons in the CNS [93], thus enabling the peptidergic neurons to respond to ETH (Figure 1b). Elevated ecdysone levels in the haemolymph prevent the release of ETH from the Inka cells [90,92]; however, a subsequent decrease in ecdysone levels then induces expression of the orphan nuclear receptor βFTZ-F1 in Inka cells, which in turn triggers the release of ETH into the haemolymph [94]. Secreted ETH remotely acts on ETHR in multiple peptidergic neurons in the CNS, which sequentially respond to ETH and generate the behavioural sequence observed during ecdysis [95–98] (Figure 1b). Peptidergic cells in peripheral tissues and the CNS thus coordinate their response to ecdysone, resulting in a highly integrated and complex behavioural sequence mediated by Inka cell-derived ETH (Figure 1b).

Eclosion hormone (EH), a peptide hormone produced by a pair of neurosecretory cells called Vm neurons in the CNS [99–103], is known to be a primary target of ETH in the induction of ecdysis behaviour [95–98]. The EH-ETH feedback loop between the CNS and Inka cells plays a critical role in regulating moulting [104,105]. A recent study by Scott et al. have used a newly generated *EH-Gal4* driver and EH antibody to show that, in addition to the Vm neurons, EH is also expressed in peripheral cells, including those located on segmental tracheal branches proximal to the ETH-expressing Inka cells [106]. Moreover, peripherally derived EH seems to be essential in regulating larval moulting, suggesting that the EH-ETH feedback loop may also function between peripheral tissues during larval moulting behaviour [106].

In higher dipterans, pupariation (puparium formation) is a stereotypic behavioural sequence that includes body contraction and glue-spreading behaviours [107–109]. Although the pupariation process is known to be triggered by ecdysone at the end of the final larval stage [108,110], the underlying molecular mechanisms are not well understood. A recent study in *Drosophila* showed that Dilp8 produced in the epidermis plays a central role in regulating pupariation behaviour [52]. Ecdysone triggers transient expression and secretion of Dilp8 from the epidermal cells (Figure 1a). Secreted Dilp8 acts on Lgr3-expressing neurons in the CNS to regulate body contraction and glue-spreading behaviours during pupariation (Figure 1a). As mentioned above, when imaginal disks grow abnormally in third instar larvae, imaginal disk-derived Dilp8 delays the onset of pupariation by antagonising ecdysone production [50,51]. However, at the onset of pupariation, epidermis-derived Dilp8 acts in a spatially and temporally independent manner to promote pupariation behaviour [52]. The Lgr3-expressing neurons responsible for the pupariation behaviour are different from those that inhibiting ecdysone production (Figure 1a). Although the underlying mechanisms whereby Lgr3-expressing neurons in the CNS regulate pupariation behaviour remain unclear, the newly discovered ecdysone-inducible peptide derived from peripheral tissues that regulates insect innate behaviour, in addition to ETH, emphasises the importance of peripheral tissue-derived peptide hormones in controlling innate behaviours during insect development.

## 3. Fat body-derived peptide hormones: nutrition-dependent responses

**Figure 2.**
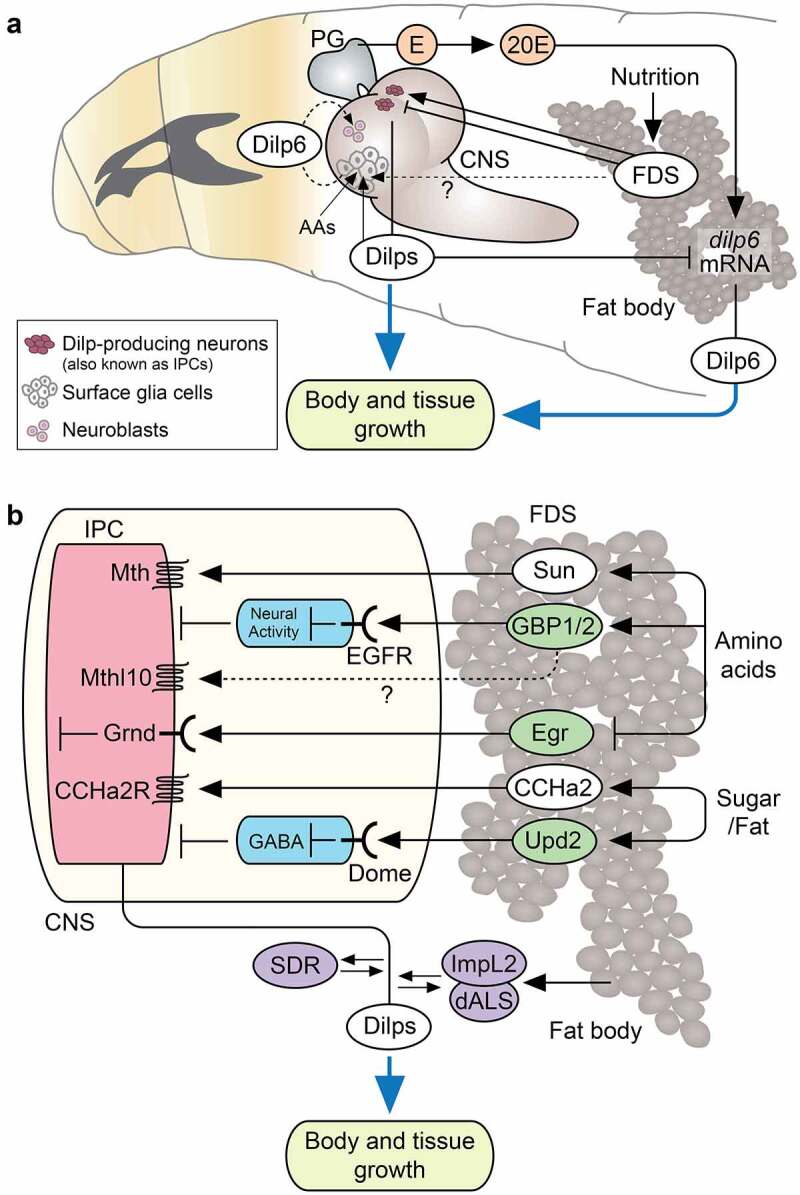
Fat body-derived signals regulating body and tissue growth. (a) *Drosophila* insulin-like peptides (Dilps) from the brain insulin-producing cells (IPCs) and Dilp6 from the fat body act as a main regulator for body and tissue growth during development. surface glia-derived Dilp6 regulates neuroblast reactivation in a nutrient-dependent manner. (b) Action of fat body-derived signals (FDSs) on IPCs for releasing Dilps into circulation during *Drosophila* larval development. different types of peptide hormones (white) and cytokines (green) coordinate IPC activities in response to multiple nutritional cues. circulating Dilp activity is further regulated by several binding proteins in the haemolymph (purple). Abbreviations; E, ecdysone (an immediate precursor of a biologically active ecdysone); 20E, 20-hydroxyecdysone (a biologically active ecdysone); PG, prothoracic gland; CNS, central nervous system; AAs, amino acids; Sun, Stunted; GBP1/2; growth blocking peptide 1 and -2; Egr, Eiger; CCHa2, CCHamide-2; Upd2, Unpaired-2; Mth, Methuselah; EGF, epidermal growth factor receptor; Mthl10, Methuselah-like 10; Grnd, Grindelwald; CCHa2R, CCHa2 receptor; Dome, Domeless; GABA, Gamma-aminobutyric acid; SDR, secreted decoy of InR; ImpL2, imaginal morphogenesis protein-Late 2; dALS, *Drosophila* acid labile subunit.

Insects store nutrients in a specialised organ called the fat body, which is equivalent to the liver and adipose tissue in vertebrates [111–113]. In addition to its function as a nutrient storage organ, fat body acts as a central organ that regulates growth and metabolism by communicating the organism’s nutritional status to other organs or tissues [4,13,25,114,115]. Fat body-derived secretory factors, including hormones and cytokines, act as endocrine signals that mediate communication between multiple organs through circulation. Recent studies in *Drosophila* have revealed the existence of multiple fat body-derived signals (FDSs), which are humoral factors, including many peptide hormones that transmit nutritional status to other organs (Figure 2a, b).

The idea of FDS in *Drosophila* originated from *ex vivo* fat body culture experiments that suggested the existence of unknown mitogenic factors secreted from the fat body during larval development [116,117]. In particular, Britton and Edgar’s co-culture experiments using the fat body and other organs suggested that the fat body secretes humoral factors that promote division of brain neuroblasts and imaginal disk cells in a nutrient-dependent manner [117]. An important milestone in this field was the characterization of Dilps produced in the brain IPCs as nutrient-dependent growth regulators in *Drosophila* [44,46,47]. IPCs predominantly produce Dilp2, −3, and −5, and genetic ablation of these cells results in a reduced larval growth rate with elevated haemolymph sugar levels [47]. Subsequent studies have further established that nutritional status is sensed primarily by the fat body and conveyed to the brain IPCs via FDSs, which in turn controls Dilp secretion in a nutrient-dependent manner [118,119]. Several cytokines, peptide hormones, and lipoproteins as FDSs are produced in response to different nutrients [115]. Here, we mainly focus on peptide hormones that function as FDSs.

During larval development in *Drosophila*, amino acids are principal nutrients required for systemic growth. Amino acid sensing in the fat body through the target of rapamycin (TOR) signalling controls the release of several FDSs that regulate the brain IPC activity. The cytokines identified as TOR-dependent FDSs are growth blocking peptides (GBP1 and −2) [120] that are structurally related to epidermal growth factor (EGF) [121,122], and Eiger (Egr) [123], a *Drosophila* tumour necrosis factor (TNF)-α homologue [124,125]. GBPs are produced in the fat body in an amino acid-dependent manner [120], and they activate EGFR expressed in a pair of intermediate inhibitory neurons called IPC-connecting neurons (ICNs), which have direct synaptic connections with the IPCs [126] (Figure 2b). Under normal food conditions, GBP-dependent activation of EGFR signalling inhibits ICN’s neuronal activity, relieving ICN-mediated inhibition of Dilp secretion from IPCs. However, under low amino acid conditions, activated ICNs inhibit Dilp secretion from the IPCs [126]. In contrast, other groups reported that GBP functions as a ligand for GPCR called Methuselah-like 10 (Mthl10) [127,128], which is expressed in adult brain IPCs [127], suggesting that GBP may also act directly on IPCs (Figure 2b). Another cytokine FDS, Egr, directly inhibits Dilp production in response to downregulation of the TOR pathway in the fat body [123] (Figure 2b). Notably, release of Egr from the fat body into the haemolymph is induced by cleavage of the transmembrane domain of Egr through induction of a TNF-α converting enzyme (TACE) under protein restriction [123]. Secreted Egr acts on IPCs via an Egr receptor called Grindelwald [129], which in turn activates the c-Jun N-terminal kinase (JNK) pathway and suppresses *dilp2* and *−5* expression in the brain IPCs [123]. The suppression of *dilps* expression by the JNK pathway in the brain IPC is consistent with previous reports [130,131].

Stunted (Sun) is a previously uncharacterised peptide hormone that controls Dilp secretion from IPCs [132]. Sun is an epsilon subunit of the mitochondrial F1F0-ATP synthase complex V, which is necessary for ATP synthesis [133]. In addition to its role in mitochondria, Sun also independently functions as a ligand for the GPCR Methuselah (Mth) [134]. Secretion of the Sun from the fat body is tightly regulated by the amino acid-sensitive machinery involved in TOR signalling. Once secreted into the haemolymph, Sun acts on Mth expressed in IPCs to induce Dilp secretion [132] (Figure 2b). Therefore, Sun functions as an insulinotropic, fat body-derived peptide hormone that is released in response to amino acids (Figure 2b). Furthermore, a recent study showed that the fat body-derived Sun contributes to sex-dependent phenotypic plasticity in *Drosophila*. Female-specific upregulation of insulin/IGF signalling activity during growth in females with larger body sizes depends on the up-regulation of *Sun* in the fat body [135]. This observation indicates that, in addition to its amino acid-dependent role in growth regulation, Sun also contributes to sexual dimorphism in growth regulation during larval development.

Sugars and lipids also modulate the brain IPC activity by stimulating release of the leptin-like cytokine Unpaired-2 (Upd2) from the fat body [136]. Upd2 activates its receptor Domeless (Dome) expressed in GABAergic neurons, which have direct synaptic connections with IPCs (Figure 2b). Under a normal food condition, Upd2-dependent activation of the JAK/STAT signalling inhibits GABAergic neuronal activity, relieving their inhibitory effect on the IPCs and triggering the secretion of Dilps into the haemolymph [136] (Figure 2b). Furthermore, the fat body-derived peptide hormone CCHamide-2 (CCHa2) regulates Dilp secretion in a sugar-dependent manner [137]. *CCHa2* expression in the fat body is reduced by starvation and promoted by glucose refeeding. The *CCHa2 receptor* (*CCHa2-R*) is primarily expressed in the CNS, including in IPCs. The CCHa2 peptide activates a calcium-activated reporter in IPCs in *ex vivo* cultured brains. Thus, CCHa2 is a fat body-derived peptide hormone that primarily responds to sugars and coordinates systemic growth in a nutrient-dependent manner [137] (Figure 2b).

In addition to nutritional availability, various environmental factors, such as oxygen availability, influence larval systemic growth via the fat body. Indeed, hypoxia leads to smaller body size by reducing their systemic growth rate in *Drosophila* [138,139]. This response to oxygen in the fat body requires the transcription factor called hypoxia-inducible factor 1 alpha (HIF-1a) that functions in a conserved major oxygen-sensing pathway [140]. Although HIF-1a-dependent FDS(s) seem to inhibit expression and secretion of Dilps from IPCs [140], the molecular nature of such FDS(s) remains unknown.

Regulation of the brain IPC activity by FDSs has also been observed in adults. Female-specific independent of transformer (FIT) functions as a fat body-derived peptide involved in regulating protein feeding through the brain IPCs [141]. FIT is produced and secreted from the fat body upon protein feeding and induces Dilp secretion from the IPCs. Consequently, the protein intake is suppressed, suggesting that FIT is a negative regulator of food intake that conveys satiety signals. However, it is still unclear which receptors FIT acts on, and through which mechanism it exerts its insulinotropic effect [141]. Altogether, the fat body functions as a vital sensing organ for environmental conditions such as nutrients and oxygen and signals environmental states to multiple organs through FDSs, which include a variety of peptide hormones.

The fat body produces two Dilp-binding proteins, Imaginal morphogenesis protein-Late 2 (ImpL2) and *Drosophila* acid labile subunit (dALS, also known as Convoluted) that regulate the activity of circulating Dilps [142,143] (Figure 2b). These two proteins form a trimeric complex with Dilps in the haemolymph and inhibit their binding to the InR [142–144]. Therefore, knockdown or mutation of either *ImpL2* or *dALS* results in an overgrowth phenotype, whereas overexpression of either *ImpL2* or *dALS* inhibits body growth during development [142,143]. Since ImpL2 protein expression is upregulated during starvation [142], the nutrient-dependent function of Dilp is tightly regulated by the FDS not only at the secretory level but also in the circulating haemolymph. In addition to ImpL2 and dALS, a secreted decoy of InR (SDR) that is structurally similar to the extracellular domain of InR can directly interact with several circulating Dilps to antagonise their activity during larval development [144] (Figure 2b).

ILPs produced in the brain IPCs play an essential role in nutrition-dependent growth during the feeding period of larvae and adults, whereas IGF-like peptides derived from the fat body is important in regulating growth during the non-feeding period. An IGF-like peptide was first discovered in *Bombyx* as a fat body-derived growth factor with characteristics similar to those of mammalian IGFs [145,146]. Dilp6 was subsequently characterised as an IGF-like peptide in *Drosophila* [48,49]. Expression of *dilp6* is induced via an inhibitory component of the insulin/IGF signalling, forxhead box, sub-group O (FoxO), upon amino acid starvation in the fat body [49], which indicates a negative feedback regulation of *dilp6* expression by circulating Dilps (Figure 2a). Expression of *dilp6* in the fat body is also induced by ecdysone during the wandering and pupal stages after cessation of feeding [48,49] (Figure 2a). Adult body size of *dilp6* mutants is about 15% smaller than that of wild-type flies, despite no apparent growth defects during the larval feeding stage [48,49]. This observation indicates that Dilp6 from the fat body plays an essential role as an ecdysone-dependent systemic growth regulator during metamorphosis (Figure 2a). Fat body-derived Dilp6 also functions in adults, wherein it regulates insulin/IGF signalling in the hepatocyte-like oenocytes to control lipid accumulation [147]. Dilp6 from the fat body is also involved in lifespan, thermoregulation, and immune response [148–150], reflecting its wide range of essential functions similar to mammalian IGFs.

Notably, an IGF-like Dilp6 is also produced by the surface glial cells that form the blood–brain barrier (BBB) of the CNS [151–154]. Although *dilp6* expression in the fat body is negatively regulated by insulin/IGF signalling, glial *dilp6* expression is positively regulated by nutritional signals, including insulin/IGF signalling and Tor signalling, via FDS [151,152,154]. Nutrition-dependent release of Dilp6 from surface glia acts on InR expressed in the neuroblasts, the neural stem cells in *Drosophila*, which in turn induces neuroblast reactivation [151–153] (Figure 2a). As noted above, Britton and Edgar’s co-culture experiments with the fat body and CNS suggested that FDS promotes neuroblast reactivation in a nutrient-dependent manner [117]. Surface glia-derived Dilp6 thus serves as a key relay molecule in the nutritional control of neuroblast proliferation in the CNS, which is isolated from systemic signals by the BBB.

## 4. Gut-derived peptide hormones: feeding and energy metabolism

**Figure 3.**
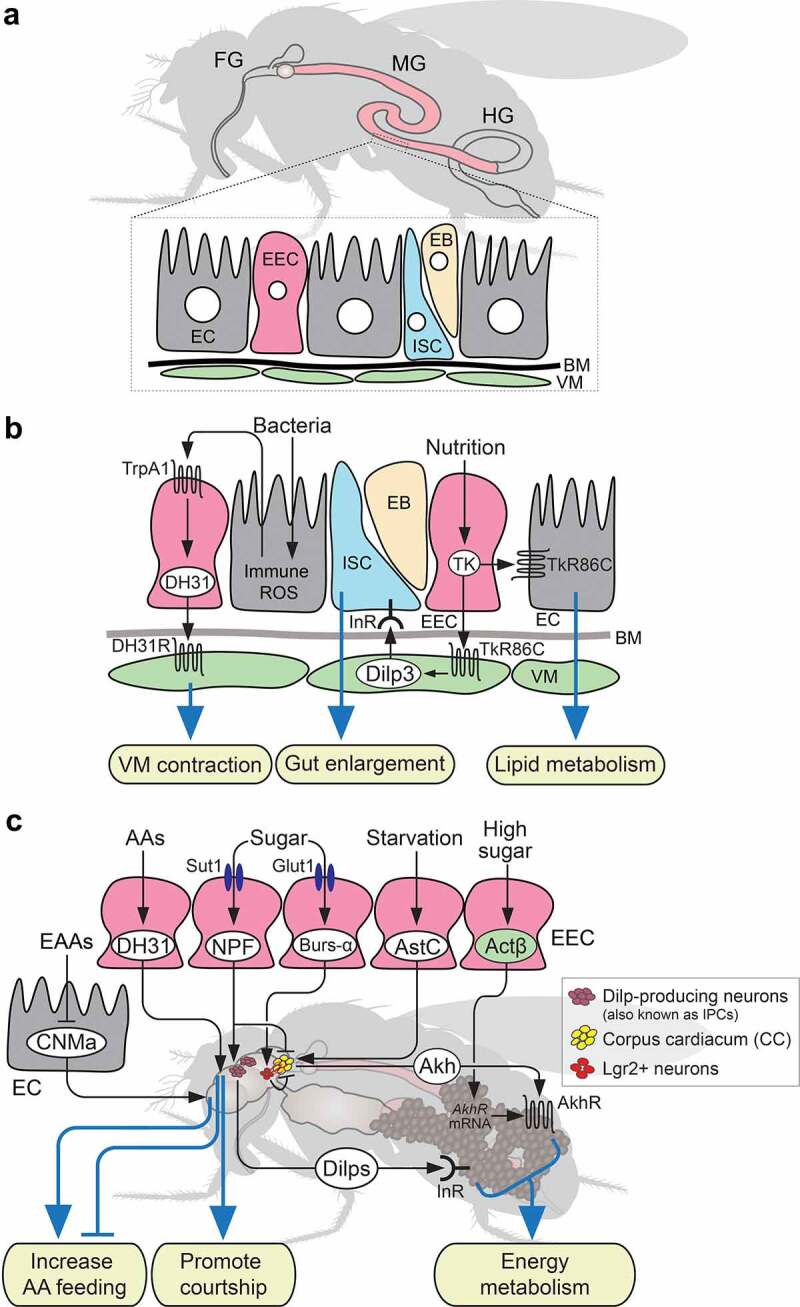
Interorgan communication by gut-derived peptide hormones in response to internal nutritional cues in adults. (a) Schematic diagram of *Drosophila* adult gut consisting of the foregut (FG), midgut (MG), and hindgut (HG). The gut epithelium consists of different types of cells: enterocytes (EC), enteroendocrine cells (EEC), intestinal stem cells (ISC), enteroblasts (EB), basement membrane (BM), and visceral muscles (VM). (b) Paracrine action of gut-derived peptides regulating VM contraction by diuretic hormone 31 (DH31) in response to bacteria-inducible reactive oxygen species (ROS) from enterocyte (EC), gut enlargement by tachykinin (TK) via *Drosophila* insulin-like peptide 3 (Dilp3) from VM, and lipid metabolism in EC by TK. (c) Systemic function of enteroendocrine cell (EEC)- and EC-derived peptide hormones (white) and cytokines (green). Abbreviations; TrpA1, transient receptor potential cation channel A1; DH31R, DH31 receptor; TkR86C, tachykinin-like receptor at 86C; AAs, amino acids; EAAs, essential amino acids; Sut1, sugar transporter 1; Glut1, glucose transporter 1; CNMa, CNMamide; NPF, neropeptide F; Burs-α, bursicon-α; AstC, allatostatin C; Actβ, activinβ; Akh, adipokinetic hormone; Dilps, *Drosophila* insulin-like peptides; AkhR, Akh receptor; InR, insulin-like receptor; Lgr2, leucine-rich repeat-containing G protein-coupled receptor 2.

The gut, or intestine, is an essential organ for food digestion, nutrient absorption, and energy homoeostasis [155–158]. Similar to mammals, the *Drosophila* gut has morphologically and functionally distinct regions: the foregut, midgut, and hindgut [157,158] (Figure 3a). The adult midgut can be further subdivided into these six regions: R0 (also known as proventriculus), R1 and R2 (anterior midgut), R3 (middle midgut and copper cell region), and R4 and R5 (posterior midgut) [159]. Furthermore, the epithelial monolayers of R1-R5 consist of different types of cells: multipotent intestinal stem cells (ISCs), progenitor enteroblasts (EBs), secretory EECs, and absorptive enterocytes (ECs) [157] (Figure 3a). The gut epithelium is surrounded by visceral muscles (VMs) and the basement membrane (BM) [157] (Figure 3a). Among these cells, the EECs function as sensors of internal gut environment, such as dietary nutrients and microbiota-derived metabolites, that secrete peptides and cytokines and regulate various physiological processes [158,160–163].

In *Drosophila*, at least 15 peptides are reported to be expressed in the gut EECs: allatostatin-A (AstA), AstC, Bursicon-α (Burs-α), crustacean cardioactive peptide (CCAP), CCHamide-1 (CCHa1), CCHa2, diuretic hormone 31 (DH31), glycoprotein hormone beta 5 (Gpb5), ion transport peptide (ITP), MIP/AstB, neuropeptide-like precursor 2 (Nplp2), neuropeptide F (NPF), orcokinin, short NPF (sNPF), and tachykinin (TK) [81,164–171]. All these EEC-derived peptides are produced in a region-specific manner in the gut, and some of them are co-expressed in the same cells [81,165,169–171]. As most of the EEC-derived peptides are also produced in the CNS, they were originally considered to function mainly within the gut as autocrine or paracrine factors. Indeed, several EEC-derived peptides act locally to regulate gut motility in VMs [172–175], proliferation of ISCs [167,176,177], and lipid metabolism in ECs [178,179] (Figure 3b). However, recently, it has become evident that EEC-derived peptides act as endocrine factors for interorgan communications and have independent functions from those produced in the CNS.

While it is not yet clear whether all EEC-derived peptides are secreted into the haemolymph, it appears that at least some peptides are indeed secreted into the haemolymph. A previous study using the locust and cockroach showed that the gut EECs release TK by depolarization, and the TK level in the haemolymph increases after starvation [180], suggesting that EECs can release peptides into circulation. In *Drosophila*, direct evidence has shown that EEC-derived Burs-α and DH31 can be detected in the circulating haemolymph [181,182]. Moreover, combinations of genetic experiments strongly suggest endocrine effects of several EEC-derived peptides on remote target organs or tissues such as the CNS, corpus cardiacum (pl. corpora cardiaca; CC), and ovary [183–187].

Several EEC-derived peptide hormones regulate energy homoeostasis by acting remotely on other organs. One such peptide is Burs-α, which is systemically secreted from the gut EECs in a dietary sugar-dependent manner [181]. EEC-derived Burs-α acts on its receptor, Lgr2 (also known as Rickets), expressed in specific neurons in the CNS and enteric neurons. Notably, Lgr2-expressing neurons form direct synaptic connections with the specialised neurohemal organs called the CC, which produce adipokinetic hormone (Akh), and negatively regulate Akh secretion (Figure 3c). Akh is a primary insect peptide hormone that is functionally equivalent to mammalian glucagon [188–191]. Akh acts on the Akh receptor (AkhR) predominantly expressed in the fat body to mobilise stored lipids and carbohydrates under energy-demanding conditions in the adult flies [192–195]. Therefore, EEC-derived Burs-α remotely regulates energy metabolism in response to feeding by inhibiting Akh production via the CC-innervating neurons [181] (Figure 3c).

EEC-derived NPF and AstC also directly act on the CC to regulate secretion of Akh [186,187] (Figure 3c). Dietary sugars positively regulate production and secretion of NPF from the gut EECs, whereas AstC secretion is induced by nutrient restriction (Figure 3c). Although both the NPF receptor (NPFR) and AstC receptor 2 (AstC-R2) are expressed in the CC, NPF and AstC have an inhibitory and facilitatory effect on secretion of Akh, respectively [186,187] (Figure 3c). NPFR is also expressed in the brain IPCs, where it facilitates secretion of Dilps [186] (Figure 3c). Therefore, EEC-derived NPF, in response to dietary sugars, regulates the balance between Dilps and Akh, promoting assimilation in peripheral tissues, such as the fat body [186]. The action of EEC-derived NPF resembles that of the mammalian enteroendocrine hormone glucagon-like peptide 1 (GLP-1). GLP-1 is known to induce insulin secretion by stimulating β-cells in pancreatic islets, whereas it suppresses glucagon secretion in α-cells in a dietary sugar-dependent manner [196,197].

Mechanisms of dietary nutrient sensing by EECs differ among the gut region and types of EECs. For example, Burs-α and NPF are produced in EECs in the posterior and anterior midgut, respectively. Dietary sugars induce production and secretion of Burs-α and NPF through different solute carrier family 2 (SLC2) sugar transporters, Glut1, and Sut1, respectively [181,186] (Figure 3c). In addition, TK-, and DH31-producing EECs, which express many gustatory receptors (Gr), appear to be activated by dietary proteins and amino acids [198,199]. Indeed, a recent report showed that dietary amino acids activate DH31-producing EECs, thereby increasing DH31 levels in the haemolymph in adult males [182]. The secreted DH31 remotely acts on the DH31 receptor (DH31R) expressed in the two populations of neuropeptide-producing cells in the CNS to regulate courtship and protein feeding (Figure 3c). DH31R expressed in the AstC-producing cells suppresses protein feeding, whereas DH31R in the Corazonin-producing cells promotes courtship behaviour. Therefore, the gut EEC-derived DH31 orchestrates the acute transition from feeding to courtship in adult males in response to dietary amino acids [182]. Together with the sugar-dependent production of EEC-derived peptides described above, these findings indicate that, as in mammals, peptide hormone release from the gut EECs is modulated in response to multiple nutrients in a gut region- and EEC type-specific manner.

The EECs also release cytokines that regulate energy homoeostasis. In *Drosophila* larvae, cytokine activin-β (Actβ) is crucial in adapting to excessive amounts of glucose in the diet [200]. EEC-derived Actβ remotely acts on the TGF-β receptor Baboon in the fat body and promotes carbohydrate metabolism by stimulating transcription of the *AkhR*, allowing adaptation to high-carbohydrate diets [200] (Figure 3c). Therefore, as a more acute response to the dietary nutrition in the gut, multiple EEC-derived peptide hormones and cytokines convey dietary information to other organs, resulting in regulation of energy metabolism.

Peptide hormones and cytokines are also produced by other types of cells in the gut. In adult *Drosophila*, gut size changes considerably in response to dietary nutrients and starvation, triggered by the activation of ISC division by the paracrine effect of Dilp3 derived from the VMs [201] (Figure 3b). The Dilp3 production in VMs is regulated by the paracrine action of TK from EECs in a nutrient-dependent manner [176] (Figure 3b).

Furthermore, peptide hormones and cytokines from ECs play an essential role in regulating feeding and metabolism. The EC-derived amidated peptide hormone, CNMamide (CNMa), regulates the feeding preference of essential amino acids (EAAs) [202] (Figure 3c). When proteins or EAAs are depleted, ECs in the anterior midgut express *CNMa* via the amino acid sensing pathway. Notably, ECs can also sense the microbiota-derived metabolites, which increase or decrease depending on the concentration of EAAs in the diet [202]. The secreted CNMa acts on the CNMa receptor (CNMaR) in the CNS to promote protein feeding (Figure 3c). Therefore, gut ECs can sense dietary amino acid information and use it to remotely control feeding behaviour through CNMa. The function of EC-derived CNMa resembles that of the peripherally derived fibroblast growth factor 21 (FGF21) in mammals, which can signal to the brain to control feeding behaviour [203–205]. Another recent study identified zinc-sensing ECs that support vigorous feeding in *Drosophila* larvae [206]. The zinc-gated chloride channel called Hodor activates TOR signalling in ECs, leading to increased food intake and secretion of Dilps from the brain IPCs through systemic humoral signals that are yet to be identified. These findings suggest that in addition to EECs, ECs serve as major sensor cells that respond to dietary nutrients and microbiota-derived metabolites in the gut.

## 5. Peripherally derived peptide hormones: post-mating responses and reproductive physiology

**Figure 4.**
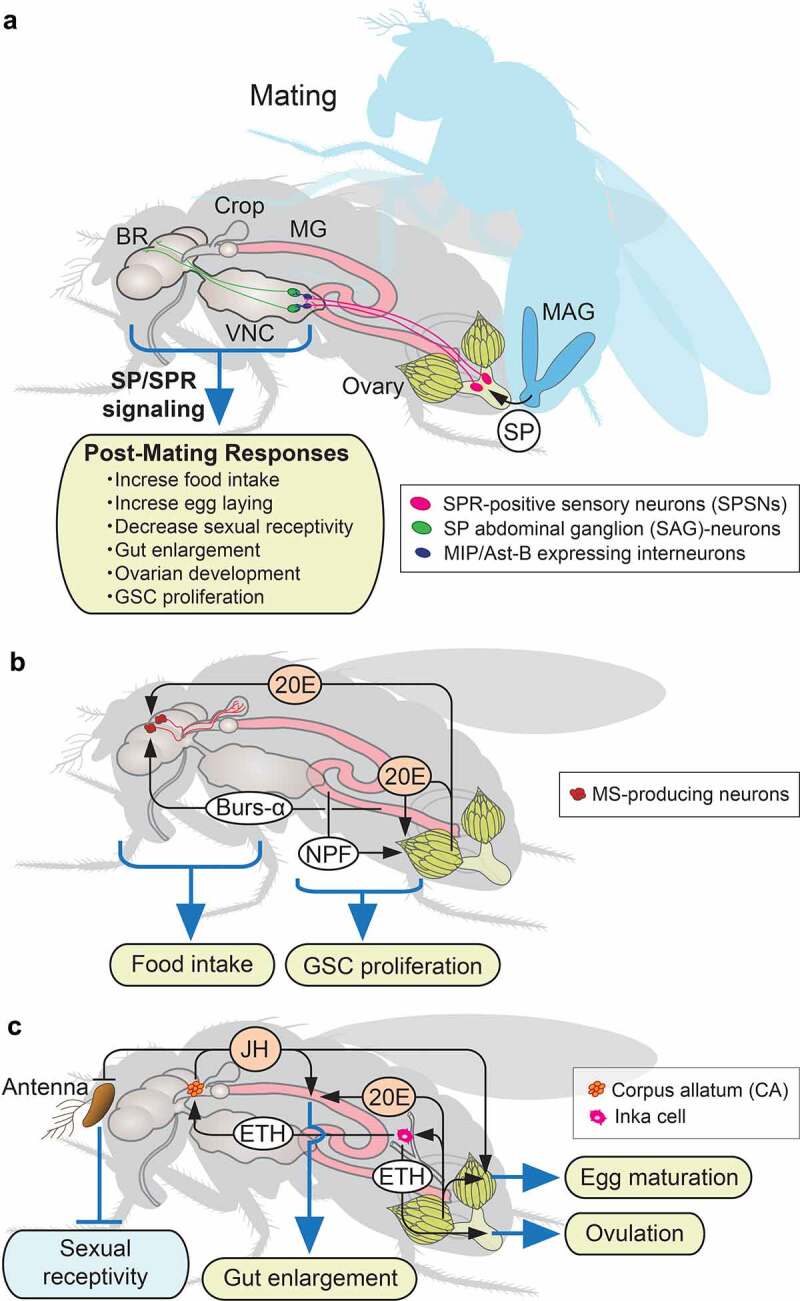
Interorgan communication by peripherally derived peptide hormones regulating post-mating response and reproduction.(a) Schematic diagram of *Drosophila* post-mating responses triggered by sex peptide (SP)/SP receptor (SPR) signalling relay from the male accessary gland (MAG) to female central nervous systems. (b) Systemic function of 20-hydroxyecdysone (20E, active form of ecdysone) and EEC-derived peptide hormones, bursicon-α (Bur-α) and neropeptide F (NPF) upon post-mating responses. (c) Systemic function of ecdysis-triggering hormone (ETH) from Inka cells in regulating egg maturation and ovulation via juvenile hormone (JH) and octopamine release upon post-mating responses. Abbreviations; BR, brain; VNC, ventral nerve cord; MG, midgut; GSC, germline stem cells.

In many insect species including *Drosophila*, mating triggers a switch in processes involved in reproduction and fertility, inducing substantial behavioural and physiological changes in females. This change is primarily mediated by accessory gland proteins (Acps), which are transferred from males to females through the seminal fluid upon mating [207–209]. In *Drosophila*, sex peptide (SP or Acp70A) is the best-characterised Acp produced only in the adult male accessory gland (MAG) [210–212] (Figure 4a). After mating, SP binds to spermatozoa and is retained in the reproductive tract of mated females for several days. SP is gradually released by proteolysis [213], and the released SP then binds to SPR in a small number of SPR-positive sensory neurons (SPSNs) located on the female reproductive tract [214–217] (Figure 4a). SPSNs innervate the uterine lumen and send afferent axons into the abdominal ganglion (AG) in the ventral nerve cord (VNC) (Figure 4a). SPSNs further relayed the SP/SPR signal to the brain dorsal protocerebrum region via SP abdominal ganglion (SAG) neurons [218] (Figure 4a). This SP/SPR signalling relay from the SPSNs to the SAG neurons is mediated by MIP/AstB-expressing female-specific interneurons located in the AG [80] (Figure 4a). SPR is also expressed in the CNS [214], and SP is considered to circulate in the haemolymph in mated females [219]. Therefore, SP may act as a peptide hormone to activate SPR in the CNS. Transmission of SP/SPR signalling from the reproductive tract to the brain leads to multiple post-mating responses, including increased food intake, reproductive maturation, and decreased sexual receptivity through downstream neuronal circuits [220,221] (Figure 4a). SPR can also be activated by MIP/AstB, another ligand for SPR [222,223]. As MIP/AstB is a well-conserved peptide in insects and the orthologue of the SP found only in a subgroup of closely related *Drosophilidae*, it is considered as an ancestral ligand for SPR [72,222,223]. The pheromonostatic peptide (PSP) produced in the MAG in the corn earworm moth *Helicoverpa zea* is also a peptide similar to MIP [224]. PSP inhibits female pheromone biosynthesis after mating, suggesting an evolutionary relationship between *Helicoverpa* PSP and *Drosophila* SP for inducing post-mating responses.

After mating, SP/SPR signalling orchestrates multiple physiological changes in several organs, including the reproductive organs and gut. These physiological changes are regulated primarily by endocrine hormones. It is known that mated females retain more germline stem cells (GSCs) than virgin females in *Drosophila* [225–228]. Mating-induced increase in GSCs requires two endocrine hormones, ecdysone and NPF [185,225] (Figure 4b). After mating, ecdysone biosynthesis in the ovary and NPF secretion from gut EECs are promoted in response to SP/SPR signalling [185,225,229]. These hormones activate EcR and NPFR in ovarian somatic cells, where they enhance bone morphogenetic protein (BMP) signalling levels in the GSCs, promoting their proliferation [185,225]. EEC-derived NPF thus has diverse functions, regulating energy metabolism in response to dietary nutrients [186] (Figure 3b) and inducing GSC proliferation in post-mating responses [185] (Figure 4b).

The post-mating increase of ecdysone can also induce behavioural changes through the CNS [230,231]. After mating, ecdysone from the ovary modulates mating-stimulated increases in food intake [230]. A recent study showed that ecdysone acts on myosuppressin (MS)-producing neurons in the PI region of the brain, along with the gut EEC-derived peptide hormone Burs-α [231] (Figure 4b). MS-producing neurons innervate directly onto the crop, a gastric-like organ, and regulate muscle contraction of the crop in response to ecdysone and Burs-α, resulting in an increase in food intake [231]. Therefore, ecdysone and gut EEC-derived peptide hormones coordinate multiple physiological changes in response to mating stimuli (Figure 4b).

In addition to ecdysone, mating triggers juvenile hormone (JH) biosynthesis and its release into the haemolymph [232–235]. JH is a sesquiterpenoid synthesised in the endocrine gland called the corpus allatum (pl. corpora allata; CA) [236]. Although JH has primary roles in regulating metamorphosis during development, it further regulates various aspects of reproductive maturation in adult stages of many insect species [237,238]. In *Drosophila* females, reduction in JH levels by CA ablation or mutation of *JH acid methyltransferase* (*jhamt*), the gene encoding a regulatory JH biosynthetic enzyme, leads to impaired egg maturation, resulting in reduced fertility [239–241] (Figure 4c). Moreover, mating-induced JH reduces sensitivity of olfactory neurons in females to male pheromones, resulting in increased preference for pheromone-rich males [242]. Although JH is known for regulating the onset of mating after eclosion [243,244], desensitization of olfactory neurons to male pheromones by JH suggests that JH is also involved in the decreased sexual receptivity after mating (Figure 4c).

Importantly, JH production and release from the CA is regulated by the peripherally derived peptide ETH [245] (Figure 4c). As mentioned earlier, ETH from epitracheal Inka cells acts on several peptidergic neurons in the CNS to trigger ecdysis behaviour during development [83] (Figure 1b). In adults, however, Inka cell-derived ETH remotely acts on the CA as an allatotropin to promote JH release into the haemolymph [245]. Disruption of ETH signalling in adults significantly reduces JH levels, resulting in reduced egg maturation and fertility (Figure 4c). ETH also activates octopaminergic neurons of the oviduct to induce ovarian contraction and ovulation in adults [246] (Figure 4c). Thus ETH, previously known for its function in regulating ecdysis behaviour during development, appears to also play critical roles in regulating egg maturation and ovulation via JH and octopamine release in adults (Figure 4c).

The gut of females also exhibits significant morphological changes after mating, mainly through ecdysone and JH (Figure 4c). *Drosophila* females enlarge their gut to facilitate digestion and absorption as their food intake increases after mating [247,248]. To this end, mating stimuli activate female gut ISC proliferation through following mechanisms: first, cell-autonomous sex determinants expressed in the ISCs cause the female ISCs to be more mitotically active than those of males [249]. Second, post-mating elevation of ecdysone and JH further increases ISC proliferation in females, amplifying gut sexual dimorphism [247,250,251]. Mating-induced changes are also observed in the gut EC. In mated females, elevated JH remodels the gut EC, resulting in enhanced reproductive maturation [247].

Although signalling pathways that connect SP/SPR signalling and ecdysone/JH biosynthesis still remain largely unclear, ecdysone and JH have essential roles in post-mating interorgan communications that regulate behavioural and reproductive changes. It will be imperative to elucidate detailed the temporal changes of these post-mating hormones and how SP/SPR signalling orchestrates such hormonal fluctuations.

## 6. Significance and future issues of research on peptide hormones produced in peripheral tissues

As described in this review, many peptide hormones derived from peripheral tissues transmit their environmental and internal status to other organs and tissues, including the CNS. Some of the peptide hormones produced in peripheral tissues are also produced in neurosecretory cells or neurons in the CNS, which is particularly evident for the gut EEC-derived peptides [160]. The gut is an organ physically isolated from the circulatory system by the VMs and BM (Figure 3a), just as the CNS is isolated from the haemolymph by the BBB. It is therefore likely that the gut and CNS secrete the same peptide hormones into circulation in response to distinct stimuli that they receive using their own sensory machineries. On the other hand, once released into the haemolymph, these peptide hormones are supposed to act on the same target tissues and induce identical responses. Future comparative studies on sensory machineries that induce release of the same peptide hormones from the gut and CNS would therefore be expected to deepen our understanding of how distinct environmental and internal cues are integrated through the endocrine system.

Many peripherally derived peptide hormones signal directly to other peripheral tissues as well as to the CNS in mammals. In insects, however, most of the environmental and internal signals mediated by peripherally derived peptide hormones seems to be mainly conveyed to the CNS. This raises an important question of how peptide hormones and cytokines from peripheral tissues pass through the BBB. Recently, it has become evident that even small molecules, such as the steroid hormone ecdysone, cannot enter the CNS without a specific ecdysone transporter expressed in the BBB in *Drosophila* [252]. Therefore, it is unlikely that peptide hormones can pass easily through the BBB. One possible mechanism is transcytosis through the BBB, whereby peptide hormones are received by specific receptors or binding proteins at the surface of the BBB and transported to the other side of the cells through vesicle trafficking. This hypothesis has been proposed for transport of many peptide hormones through the BBB in mammals and other species [253,254], although available pieces of evidence are still controversial. Another possibility is that peptide hormones act through the neurohemal-releasing sites of target neurosecretory cells. Many neurosecretory cells, including Dilp-producing IPCs, extend their axonal terminals to neurohemal-releasing sites outside of the CNS to secrete peptides into the haemolymph [11]. Therefore, these sites are the most directly accessible sites for circulating peptide hormones in the haemolymph. A similar argument also applies to how peptide hormones and cytokines derived from the gut EECs and ECs can be secreted into the haemolymph through the VMs and BM that surround the gut. In insects, the internal dynamics and transport pathways of peptide hormones between different organs are still largely unknown, and this is a significant issue that needs to be addressed in the future.

In recent years, molecular genetic tools in *Drosophila* have led to a rapid progress in our understanding of endocrine hormone functions and their modes-of-action. However, there are few biochemical studies in *Drosophila*, especially related to investigating the properties and structures of secreted bioactive mature peptides in the haemolymph. During peptide production, translated prepropeptides (precursor peptides) are modified by various processing enzymes to become mature peptides [255]. However, as expression and function of these enzymes seem to vary among different types of peptide-producing cells, the structures of mature peptides may differ among different cell types even if they express the same peptide-encoding genes. For example, the *Drosophila Capability* (*Capa*) gene that encodes three mature functional peptides, Capa-1, Capa-2, and pyrokinin (PK)-1, is expressed mainly in a pair of large SEG neurons (Capa-SEG neurons) and three pairs of neurons in the VNC (Capa-Va neurons) [256–258]. Analyses of axon terminals from each neuronal group by direct mass spectrometry showed that only PK-1 is present in the terminal of the Capa-SEG neurons, while all three peptides, Capa-1, Capa-2, and PK-1, were detected in the terminal of the Capa-Va neurons [259,260]. This variation in mature peptides produced in different cells can be explained by the differences in the expression and function of processing enzymes in each neurosecretory cell. The same scenario can be applied to differences in the expression of processing enzymes in peripheral tissues. For example, mature bombyxin, an ILP in *Bombyx* expressed primarily in the brain IPCs, is secreted as a heterodimer consisting of an A- and a B-chain similar to mammalian insulin [146,261]. In contrast, the *Bombyx* IGF-like peptide, highly expressed in the fat body, is secreted into the haemolymph as a single-chain polypeptide, similar to mammalian IGFs, even though it has dibasic amino acid residues that are typically recognised by processing enzymes for cleavage [145,146]. Fat body cells probably lack the processing enzyme(s) that cleave peptides at dibasic sites, at least in the silkmoth *Bombyx*, suggesting that the mature form of ILPs may be different depending on the type of cells in which the ILP gene is expressed. Likewise, alternative splicing can produce distinct forms of peptide hormones encoded by some genes, such as *ITP* and *orcokinin* [168,262,263]. These peptide hormones that derive from the same gene may have different binding affinities to the same receptor, or they may even activate distinct receptors. Therefore, even if the same peptide hormone is considered, it is always necessary to examine the possibility that the structure and function of the mature peptides may differ depending on where they are produced.

Related to the properties and structures of secreted bioactive mature peptides, analysis of the half-life of peptide hormones and changes in their concentration in the haemolymph is also a significant issue for the future. Since peripherally derived peptide hormones are transported through the circulating haemolymph, their half-life depends partly on the structural properties and specific-binding proteins that protect them from the various peptidases present in haemolymph and on cell surfaces. Currently, valuable methods to quantitatively measure the amount of endogenous peptide hormone levels in the haemolymph remain conventional enzyme-linked immuno sorbent assay (ELISA) and Western blotting analysis. However, these methods highly depend on the quality of antibodies and require synthetic peptide standards. Furthermore, it is difficult to prepare large amount of fresh haemolymph samples from the tiny fruit fly. An innovative method has been developed to quantitatively measure the concentrations of endogenous Dilps in the haemolymph by combining genetic manipulation techniques in *Drosophila* [264]. In this method, transgenes encoding two different immuno-detectable epitope tags are inserted at both ends of the genomic region encoding the mature peptide, allowing for detecting double-tagged mature peptides by ELISA [264]. However, this method may not be useful for many peptide hormones whose C-terminus are post-translationally modified to function as bioactive mature peptides (e.g. amidated peptide hormones). Considering all these issues, developing methodologies that can comprehensively and directly analyse the structure and concentration of mature peptides from a small amount of haemolymph will be critically important in future studies.

## 7. Concluding remarks

Recent developments in cell- and tissue-specific genetic manipulation tools and advances in omics analyses have revealed that many peptide hormones produced in peripheral tissues play essential functions as signalling molecules in interorgan communication. Currently, more than 50 different neuropeptides and peptide hormones have been identified in *Drosophila*. According to the expression profiles of these peptide-encoding genes on the FlyAtlas database (http://flyatlas.org/atlas.cgi) [265], many peptides appear to be expressed in peripheral tissues, including those not described in this review. A recently published comprehensive single-cell atlas of the entire adult *Drosophila* called Fly Cell Atlas (https://flycellatlas.org/) could further elucidate peptide hormone-expressing cells at a single-cell resolution [266]. Furthermore, development and improvement of proximity ligation techniques have enabled *in vivo* secretome approaches for profiling of tissue-specific secreted proteins using various animal models [267–270], including *Drosophila* [269]. These methods may lead to the comprehensive identification of new types of peptide hormones that have not been characterised so far. The fat body-derived peptide Sun, as described in this review, is a typical example that has not been previously characterised as a peptide hormone [132]. Although physiological functions of many peptide hormones are still unknown, further development of genetic manipulation tools and biochemical analysis techniques is expected to rapidly advance our understanding of the target organs/tissues and functions of peripherally derived peptide hormones in *Drosophila*.

## Data Availability

No datasets were generated or analysed during the current study.
